# NK cell depletion delays corneal allograft rejection in baby rats

**Published:** 2010-10-02

**Authors:** Johannes Schwartzkopff, Simona L. Schlereth, Moritz Berger, Laura Bredow, Florian Birnbaum, Daniel Böhringer, Thomas Reinhard

**Affiliations:** University Eye Hospital, Killianstrasse 5, 79106, Freiburg, Germany

## Abstract

**Purpose:**

Penetrating keratoplasty has a very poor outcome compared with adults if performed in the first years of life. Rejection in these young patients occurs even in the absence of known immunological risk factors. Recently, a baby rat model was introduced and an essential contribution of natural killer (NK) cells during allograft rejection was suggested. To analyze this, NK cells were depleted in baby rats before keratoplasty.

**Methods:**

Allogeneic keratoplasty was performed between Lewis and Fisher rats. The recipient's ages were 10 and 3 weeks, respectively. NK cells were depleted by an intraperitoneal injection of a monoclonal antibody. All experiments were controlled by the injection of isotypic control antibodies and syngeneically. Survival rates were calculated and cellular infiltrates were analyzed histologically.

**Results:**

NK cell depletion did delay median graft survival times in a statistically significantly way compared with the control animals (p<0.01). At median rejection time points, macrophages, CD4^+^ T cells and CD25^+^ leukocytes infiltrated to a greater extent in the depleted recipients. No significant changes in the cell numbers of infiltrating CD8^+^ T cells were observed.

**Conclusions:**

We conclude that NK cells play a role during allograft rejection in baby rats, but their effect is replaceable. A greater infiltration of macrophages and CD4^+^ T cells suggests that they might compensate for the missing NK cells’ response in this experimental setting. Our results represent another step toward understanding the complex mechanisms of an accelerated corneal graft rejection in infant recipients.

## Introduction

Corneal transplantation has a very good outcome in terms of graft survival. In humans in low-risk situations, allograft survival rates are more than 90%. The survival rate decreases dramatically if other issues exist [1], such as ocular surface inflammation (e.g., infection, chemical burn, re-transplantation) or immune-mediated disease (e.g., allergies, rheumatologic disorders). In these high-risk patients, corneal allograft survival is reduced to 30%. A third group of patients includes young children (<3 years of age) where graft survival occurs in only 15%–35%, despite the absence of local or systemic inflammation [2-4]. Taking into consideration that the risk of rejection in solid organ transplantation in children (e.g., kidney) is not increased [5,6], this clinical observation following keratoplasty is surprising. The importance of a high success rate following infant keratoplasty is emphasized by the fact that any form of uncorrected corneal opacity in infancy leads to amblyopia. To develop effective treatment strategies that promote graft survival following infant keratoplasty and prevent the development of amblyopia, it is of intrinsic interest to understand the mechanisms of the rejection process in an infant’s eye.

To study the immune mechanisms of corneal transplant rejection, animal models are widely used. In rodent keratoplasty, CD4^+^ T cells have been shown to be key elements during immunological graft failure [7]. Also other immune factors as CD8^+^ T cells, macrophages or natural killer (NK) cells have been shown to contribute to some extent to graft failure [8-11]. Hori et al. [12] showed for the first time that the corneal epithelium plays a major role and hypothesized that it acts during the process of sensitizing the immune system toward a response. The contribution of epithelium-born antigens during the priming process was additionally proven by Saban et al. [13]. A substantial amount of corneal corneal antigen presenting cells (APC) is located within the epithelial layer, which might explain these findings. In the context of high-risk keratoplasty, preformed corneal vessels and an elevated number of corneal APC are linked to promoting a faster presentation of antigen and a faster stimulation of the alloresponse against a corneal graft [14,15].

Taken together, an allograft rejection is a multi-factorial scenario mediated by systemic and local immune factors. Moreover, its different components can compensate for each other to a certain extent. Even if a corneal allograft faces this complex system, the majority of transplants are accepted in adult humans. In short, the survival of a transplanted cornea can be traced back to the immune status of the eye [9,10]. This exceptional position in the immune system is, to a certain extent, not applicable in high-risk keratoplasty. However, little is understood about mechanisms of the ocular immune privilege in young individuals. The clinically increased rejection rate of a corneal allograft in young children suggests differences in the protective ocular immune situation: In this context changes in juvenile immune privilege, e.g., functionally different ocular APC or alterations in the components of the aqueous humor might play a role.

To analyze changes of the ocular immune privilege or related immune mechanisms in infants and their impact during infant keratoplasty, we recently introduced a baby rat model for corneal transplantation that resembles the situation in young humans: Without additional immunological differences or risk factors, young recipients of a corneal allograft show a significantly earlier rejection compared with old rats [16]. We confirmed a dominating infiltration of NK cells at all stages of the rejection period that even outlasted the actual rejection time point for grafts in young recipient animals compared with old rats [16,17]. During infancy, NK cells are a part of the innate immune system and can compensate, to a certain extent, for the missing T cell repertoire [18-20]. Our hypothesis that NK cells contribute to the process of immunological graft failure following keratoplasty in young recipient rats is therefore likely and was analyzed by depletion experiments with the baby rat model.

## Methods

### Animals and anesthesia

Inbred female Fisher (Rt1^lv1^) and Lewis (Rt1^l^) rats (Charles River, Sulzfeld, Germany) were used as donors and recipients. All animals were treated in accordance to the ARVO Statement for the Use of Animals in Ophthalmic and Vision Research. The rats were anesthetized with a short inhalation of isofluran followed by an intraperitoneal injection of a mixture of ketamin (Essex, München, Germany), xylacin (Bayer, Leverkusen, Germany) and atropin (Braun, Melsungen, Germany).

### Corneal transplantation

Orthotopic penetrating keratoplasties were performed as described [16]. Briefly, donor buttons from the central cornea were obtained using a 2.5 mm trephine and subsequently stored in conservation medium (Medium2; Biochrom, Berlin, Germany). The central cornea of a recipient was removed using a 2.0 mm trephine. The graft was fixed with eight interrupted sutures (11.0 Ethilon; Ethicon, Norderstedt, Germany). Finally, a blepharoraphy was applied for three days.

### Clinical graft assessment and rejection kinetics

All grafts were examined daily by two independent investigators for signs of opacity according to an international score [16,21]. Grafts with technical complications such as cataracts, infections, loss of the anterior chamber or massive hyphema were excluded. The time point of rejection was identified as soon as an opacity score of 4 (complete opacity) was recorded.

### NK cell depletion

Three days and then one day before the keratoplasty a monoclonal antibody (clone 3.2.3 – mouse-anti-rat CD161, provided by Bernhard Vanhove, Inserm, Nantes, France) was injected intraperitoneally to systemically deplete NK cells [22]. Control animals received an isotypic antibody. Injections were repeated every five days following the transplantation. NK cell depletion was controlled by flow cytometric analysis of blood, spleen and lymph nodes. The samples were stained with anti-CD3 FITC (clone G4.18; eBioscience - NatuTec, Frankfurt, Germany) and anti-CD161 Pe (clone 10/78; AbD Serotec, Düsseldorf, Germany) antibodies.

### Groups

Only 10-week-old (old) donor buttons were used, considering the fact that in humans the majority of grafts are derived from adults. Four different donor recipient combinations were analyzed. Group NK+ consisted of Fisher donors’ corneal buttons grafted to 3-week-old (young) Lewis recipients that were treated with an isotypic control antibody. In group NK-, young Lewis recipients were systemically depleted of their NK cells and subsequently grafted with a Fisher cornea. The corresponding syngeneic controls are indicated as syn.NK+ and syn.NK-, respectively. All groups are shown in Table 1. Additionally, old recipient Lewis rats were NK cell depleted or injected with the isotypic control antibody before keratoplasty. Each group consisted of 12 recipients.

**Table 1:** Systematic overview of transplantation groups.

**Group**	**Donor**	**Recipient**	**Treatment**
NK+	Fisher	lewis	Isotype
NK-	Fisher	Lewis	3.2.3
Syn. NK+	Lewis	Lewis	Isotype
Syn. NK-	Lewis	Lewis	3.2.3

All transplantation groups are shown systematically: If the isotypic control antibody was injected groups are named NK+. Groups where NK cells were depleted by injecting 3.2.3 (NK cell depleting antibody) are named NK-. If not mentioned an allogeneic transplantation was performed (i.e. Fisher donor and Lewis recipient); syngeneic transplantation (i.e. Lewis donor and recipient, respectively) is highlighted by adding "syn." the group name.

### Immunohistochemistry

The bulbi of six rats per group were snap frozen and cryosections were taken for immunohistological analysis [16]. Specimens were fixed with acetone. Unspecific binding was blocked by incubation in a Tris buffer containing 10% calf serum. Primary mouse and anti-rat antibodies were applied to the sections. A biotinylated rabbit anti-mouse secondary antibody was used to bind a streptavidine alcaline phosphatase. After incubation with the substrate (alkaline phosphatase kit 1; Vector, Burlingame, VT), sections were counterstained with Mayer’s hematoxylin. Positively stained cells were counted within three squares in the grafted corneal stroma by two independent investigators and calculated as mean cellular infiltrate per mm^2^.

### Antibodies for histological analysis

Primary antibodies used for immunohistochemistry were anti-CD4 (clone W3/25), anti-CD8 (clone OX-8), anti-CD161 (clone 10/78), anti-CD163 (clone ED2), anti-dendritic cells (clone OX-62) and anti-CD25 (clone Ox-39). All of these antibodies were purchased from AbD Serotec. The secondary biotinylated polyclonal rabbit and anti-mouse immunoglobulin and streptavidine alkaline phosphatase were obtained from Dako (Hamburg, Germany).

### Statistics

The time interval from corneal transplantation to the onset of rejection was analyzed using the Kaplan–Meier method and the groups were compared using the log-rank test. The densities of the infiltrating immune cells were compared statistically using the *t*-test.

## Results

### Delayed corneal allograft rejection in NK-cell-depleted young recipients

**Figure 1:**
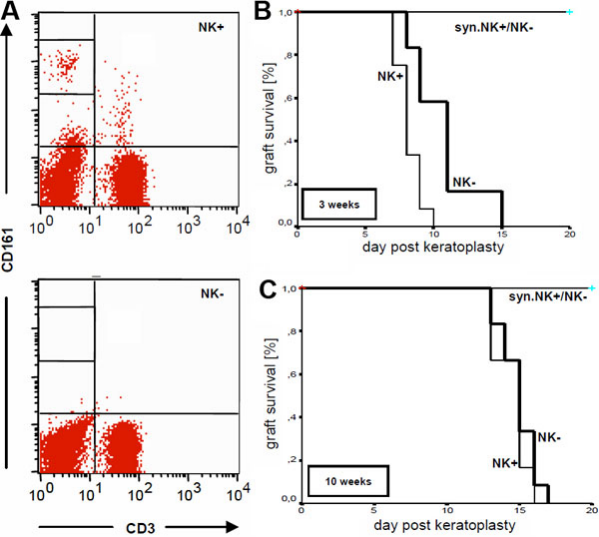
Graft survival in NK cell depleted recipients. **A**: 3-week-old (young) Lewis rats were injected twice with a NK cell depleting antibody (3.2.3). Blood was taken and analyzed by flow cytometry for the presence of CD3^+^ and CD161^+^ cells. Intraperitoneal injection of 3.2.3 lead to complete absence of CD161^+^ NK/NKT cells and did not affect cell numbers of CD3^+^CD161^-^ T cells. FACS data shown are representative for control animals that received an isotypic antibody (NK+) or 3.2.3-treated rats (NK-). **B**: After injections of either isotyp controls (NK+) or 3.2.3 (NK-) allogeneic penetrating keratoplasty was performed and the grafts were assessed clinically until rejection occurred. Albeit all recipients of both allogeneic groups rejected, animals of group NK- showed a statistically significantly delay of immunological allograft failure (p<0.01). None of the syngeinic controls (groups syn.NK+ and syn.NK-, respectively) rejected. **C**: NK cells were depleted in 10-week-old (old) Lewis rats (NK-) or isotypic antibody was injected (NK+). NK cell depletion had no effect on graft survival rates.

After two intraperitoneal injections of 3.2.3 monoclonal antibody into the Lewis rat, blood samples were taken to analyze the efficacy of the depletion. The samples were stained and analyzed by flow cytometry for the presence of CD3^+^ and CD161^+^ cells. The 3.2.3-injection resulted in a complete absence of CD3^-^CD161^+^ NK and of CD3^+^CD161^+^ NKT cells. NK cell staining is shown in Figure 1A. Cell numbers of CD3^+^CD161^-^ T cells were not affected by the treatment. Similar results were obtained for the spleens and lymph nodes. No differences in the efficacy of the treatment between young and old Lewis rats were observed (not shown). According to the protocol, young recipients were NK cell depleted (NK-) or treated with control antibodies (NK+) before keraoplasty. None of the syngeneic controls (syn.NK+ and syn.NK-) rejected a corneal allograft. Allogeneic control animals (NK+) rejected after a median time of eight days. Depletion of NK cells led to a statistically significant delay of the rejection with a median rejection time point at postoperative day 11 in group NK- (p<0.01; Figure 1B). Moreover, 3.2.3-treatment did not affect the rejection time course in old recipients (Figure 1C).

### Infiltration of CD45^+^ leukocytes and T cell subpopulations

**Figure 2:**
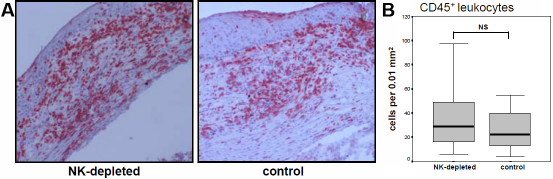
Analysis of graft-infiltrating leukocytes. At the time points of corneal allograft rejection CD45^+^ leukocytes were stained and calculated within the graft. **A**: Histological staining of graft infiltrating CD45^+^ leukocytes is shown exemplarily. **B**: Calculation from 4 representative grafts revealed no statistically significant differences in the total amount of infiltrating cells in rejected grafts of 3.2.3- and control treated recipients, respectively (NS=not significant).

**Figure 3:**
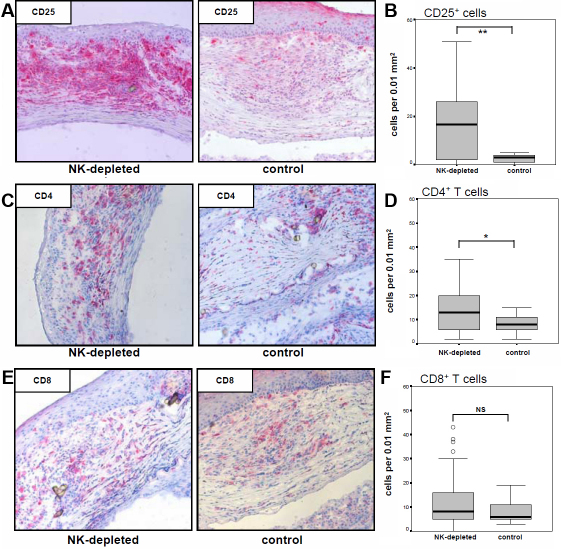
Analysis of graft-infiltrating T cells. Activated CD25^+^ T cells and CD4^+^ and CD8^+^ T cell subsets were stained at the time points of corneal allograft rejection and calculated within the graft. **A**, **C**, and **E** show representative histological staining for CD25, CD4, and CD8 in grafts of treated and control animals, respectively. CD25^+^ (**B**) and CD4^+^ (**D**) cells infiltrated to a statistically significantly stronger extent in 3.2.3-treated animals when compared to control treated animals (*p<0.01, ** p<0.01). No statistical difference was observed for CD8^+^ T cells (NS=not significant; **F**).

At the time points of rejection infiltration of CD45^+^ leukocytes was calculated within the allografts of young recipients. As shown in Figure 2A,B, no significant differences in the absolute leukocytic infiltrate were observed between control and NK cell-depleted groups. Because T cells are known to be key elements during corneal allograft failure, CD25 that is expressed on activated T cells was analyzed. In addition, CD4^+^ and CD8^+^ T cells were stained in grafts at the aforementioned time points. CD25^+^ cells were stained to a statistically significant stronger extent in NK-cell-depleted animals compared with control animals (p<0.01; Figure 3A,B). Similar results were observed for infiltrating CD4^+^ T cells (p<0.01; Figure 3C,D), whereas no significant differences for CD8^+^ T cells were shown (Figure 3E,F).

### Increase of infiltrating macrophages in NK-cell-depleted recipients

**Figure 4:**
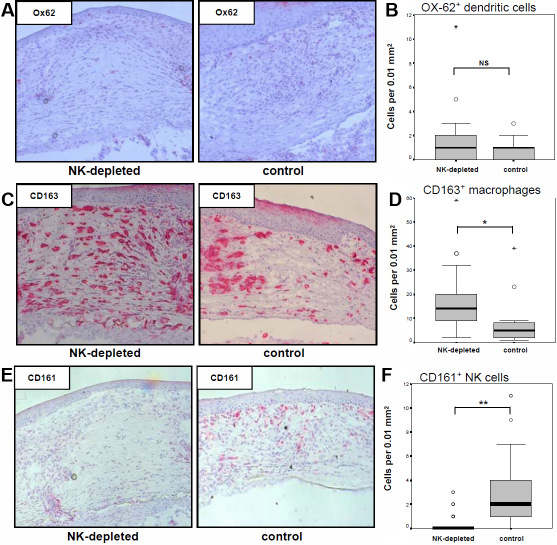
Analysis of innate immune cells into a corneal graft. Ox-62^+^ DC and CD163^+^ macrophages were stained at the time points of corneal allograft rejection and calculated within the graft. Additionally CD161^+^ cells were counted within rejected corneal allografts to finally prove the efficacy of the depletion protocol in the peripheral tissue. Representative histological staining is shown for Ox-62 (**A**), CD163 (**C**), and CD161 (**E**) in NK deficient and control animals. **B**: No statistical difference was observed for infiltrating Ox-62^+^ DC. **D**: CD163^+^ macrophages infiltrated to a statistically significantly stronger extent in 3.2.3-treated animals when compared to control treated animals (*p<0.01). **F**: No CD161^+^ cells were stained in 3.2.3-treated recipients when compared to control treated control animals (**p<0.001).

Ox-62^+^ dendritic cells (DC) and CD163^+^ macrophages were stained within corneal allografts at the time point of allograft rejection in young treated recipients. In addition, CD161^+^ cells were also calculated to finally prove their systemic absence in NK-cell-depleted animals. As shown in Figure 4A,B, NK cell depletion did not affect the infiltration of Ox-62^+^ DC. CD163^+^ macrophages infiltrated at a statistically significantly stronger level in the rejected grafts of NK-cell-depleted recipients compared with the control animals (Figure 4C,D). No CD161^+^ NK cells could be stained in rejected grafts of NK-cell-depleted Lewis recipients, whereas a substantial amount of CD161^+^ cells infiltrated in the control animals (p<0.001; Figure 4E,F).

## Discussion

In humans, corneal graft failure in children under three years of age occurs more frequently than in adults [2-4]. Because any form of uncorrected corneal opacity leads to the development of life-long amblyopia in these children, it is of great clinical interest to improve corneal graft survival in children. Recently, we introduced the baby rat model for keratoplasty that allows us to study the underlying mechanisms experimentally. Analogous to humans, young rats rejected a corneal allograft significantly earlier than did old animals. Histological analysis demonstrated a stronger infiltration of NK cells in the young rats compared with the old recipient rats [16]. We therefore hypothesized that NK cells contribute to immunological graft failure following keratoplasty in infants.

To analyze this hypothesis, NK cells were depleted in young Lewis rats before keratoplasty (Figure 1A). The systemic absence of NK cells was proven by flow cytometry of blood and lymphoid organs as well as histologically in peripheral tissues (Figure 1A and Figure 4). Importantly, no negative side effects in the T cell pool were observed, excluding that the key player for corneal graft rejection in old recipients (i.e., T cells) would also be affected. NK cell depletion lead to a significant delay of the median rejection time point compared with control-treated animals (day 11 versus day 8; Figure 1B). This finding demonstrated that NK cells did contribute to corneal graft rejection in young rats, whereas their influence on graft rejection in old recipients seemed to be negligible (Figure 1C). From mice, it is known that a missing component of their immune system is replaceable by other factors, such as macrophages, CD8^+^ T cells or complementary factors [7,8]. Depending on the conditions at the time of transplantation (i.e., low risk, high risk, type of model used, etc.), the composition of the various immune factors is variable and some components can compensate for others [9]. However, the different immune mediators can reach a critical limit that leads to rejection in diverse pathways whereby rejection kinetic is affected. A similar event seems to happen in our model and will be discussed later.

To understand compensatory mechanisms at the graft side that finally cause graft rejection in young rats in more detail, we did histological analysis of rejected grafts and compared them to the total cellular infiltration. As shown in Figure 2, the total amount of CD45^+^ leukocytes did not vary between both allogeneic groups. This finding underlines our hypothesis that the immune system is compensating for a missing component. It clearly demonstrates that a critical amount of cellular infiltration is required and is sufficient to achieve graft rejection [16]. NK cell depletion delays the time point when the critical and compensatory immunological cell infiltration is reached without preventing rejection.

We analyzed specific leukocyte populations within this immune infiltration to get more insight into the actual compensatory mechanisms that still have the capacity to reject the corneal graft. NK cell depletion led to an increase of graft infiltrating CD25^+^ cells, pointing to a contribution of T cells in the rejection process of treated rats. Only a slight increase of CD8^+^ T cells in NK-cell-depleted recipients was observed, whereas CD4^+^ T cells infiltrated to a significantly greater extent (p<0.01; Figure 3). This suggests that CD4^+^ rather than CD8^+^ T cells contribute to the immune reaction to the graft in NK-cell-deficient recipients. However, it remains unclear whether the infiltrating CD4^+^ T cells are allo-protective or allo-reactive as there are CD4^+^CD25^+^ T cell subpopulations with different effector mechanisms: On the one hand, regulatory T cells (Treg) with inhibitory functions express CD4 and CD25. A role for these Treg was proposed in pediatric liver transplantation [23] and has just recently been suggested in the mouse keratoplasty model [24]. Their contribution to a prolonged allograft survival following infant keratoplasty would therefore also be possible.

On the other hand, effector T cells also express CD4 and CD25. Their important role during corneal allograft rejection in adults is indisputable [7] and therefore is also likely in the baby rat model. It would be necessary to deplete CD4^+^ T cells alone or in combination with NK cells to understand their general contribution to corneal allograft rejection in infants. To distinguish between activated and regulatory CD4^+^ T cells, the expression of the transcription factor forkhead box protein 3 (FoxP3) that is mainly active in Treg [25] could be analyzed by rtPCR or by flow cytometry within infiltrating CD4^+^ T cells. The role of CD4^+^ lymphocytes populations is likely in our model; however, their function in the rejection process remains unclear.

Looking at infiltrating macrophages as another main arm of the innate immune system that has a stronger influence in the early times of life [26-28] revealed a significant increase of CD163^+^ macrophages in grafts of NK-cell-deficient recipients (p<0.01; Figure 4A,B). Because macrophages have been shown to affect immunological graft failure after keratoplasty in mature mice [29,30], we would suggest that they could also compensate for NK cell deficiency in infants and promote rejection. However, only depletion of macrophages alone by clodronate liposomes [31] or together with NK cell depletion would explain their role during allograft rejection in young recipients.

Finally and aside from the analysis of the infiltrate of leukocytes, it has to be taken into consideration that the depleting antibody used is directed against CD161 expressed by NK and NKT cells. NKT cells are known to contribute to the development of anterior chamber induced immune deviation (ACAID) [32,33], which is one mechanism of the ocular immune privilege that otherwise promotes the good survival rates of a corneal allograft [9,10]. In our experimental setting, both CD161^+^ cell populations are eliminated (Figure 1A). Therefore, 3.2.3-treatments could have different effects that would explain the delayed rejection: Initially, the aggressive NK cell response to an allograft in young rats would be weakened and rejection would not occur as fast as in control animals. At the same time, ACAID-promoting NKT cells would also be erased. Thereby, one mechanism of the ocular immune privilege that would otherwise promote graft survival [31,32] would be weakened as a side effect. This would explain that rejection would only be delayed but could still occur in all young recipients (Figure 1B). To exclude that NK and NKT cells act in this antagonistic way during the process of corneal graft rejection in young recipients, it would be necessary to selectively deplete every single cell population. Moreover, the effect of the treatment with 3.2.3 on the ability to develop ACAID could be analyzed in this model.

Considering these data together, we demonstrated that NK cells contribute to allograft rejection after keratoplasty in young rats. However they are obviously not solely responsible because NK-cell-deficient young recipients still reject a corneal allograft at a rate of 100%. Our results suggest that the rejection of a corneal allograft in young recipients is a multi-factorial process that involves NK cells and macrophages as parts of the innate immunity, as well as T cells of the developing adaptive immune response. Weakening one mechanism, here by the depletion of NK cells, seems to strengthen another one, e.g., macrophages and/or CD4^+^ T cells.
